# Extra‐pair paternity, breeding density, and synchrony in natural cavities versus nestboxes in two passerine birds

**DOI:** 10.1002/ece3.10163

**Published:** 2023-06-08

**Authors:** Irene Di Lecce, Charles Perrier, Marta Szulkin, Joanna Sudyka

**Affiliations:** ^1^ Institute of Evolutionary Biology Biological and Chemical Research Centre Faculty of Biology University of Warsaw Warsaw Poland; ^2^ CBGP, INRAe, CIRAD, IRD, Montpellier SupAgro University of Montpellier Montpellier France; ^3^ Institute of Environmental Sciences Jagiellonian University Kraków Poland; ^4^ Groningen Institute for Evolutionary Life Sciences (GELIFES) Groningen Netherlands

**Keywords:** cavity‐nesting birds, extra‐pair paternity, genotyping by sequencing method, natural cavity, nestbox, SNP data

## Abstract

Most of what is known about extra‐pair paternity in hole‐nesting birds derives from studies using artificial nesting sites, such as nestboxes. However, it has rarely been investigated whether inference drawn from breeding events taking place in nestboxes matches what would be observed under natural conditions, that is, in natural cavities. We here report on a variation in promiscuity in blue tits and great tits nesting in natural cavities and nestboxes in an urban forest in Warsaw, Poland. Specifically, we tested whether local breeding density, local breeding synchrony, and extra‐pair paternity (inferred from SNP data generated with a high‐throughput genotyping by sequencing method) differed between birds nesting in natural cavities and nestboxes. In both blue tits and great tits, the frequency of extra‐pair paternity was similar between the two cavity types. In blue tits, we observed shorter nearest neighbor distance, higher neighbor density, and higher synchronous neighbor density (i.e., density of fertile females) in nestboxes relative to natural cavities. No such pattern was found in great tits. Moreover, we detected a positive relationship between the proportion of extra‐pair offspring in the nest and neighbor density around the nest in blue tits. Our results revealed that the provisioning of nestboxes did not change rates of extra‐pair paternity, suggesting that conclusions drawn from nestbox studies might adequately represent the natural variation in extra‐pair matings in some species or sites. However, the observed differences in spatiotemporal components of breeding dynamics highlight the fact that these parameters should be carefully considered when comparing mating behavior across studies and/or sites.

## Graphical abstract

Extra‐pair paternity levels were similar between natural cavities and nestboxes in blue tits and great tits breeding in an urban forest in Warsaw, Poland. We observed differences in spatiotemporal parameters between cavity types in blue tits but not in great tits, namely shorter nearest neighbor distance, higher neighbor density and higher synchronous neighbor density in the nestbox plot compared with the natural cavity plot. Independently of cavity type, the proportion of extra‐pair offspring per nest increased with density of closest neighbors in blue tits.

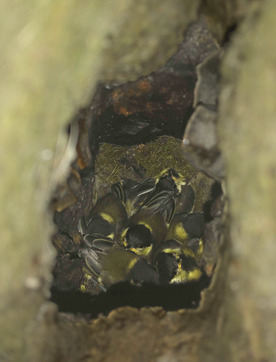

## 1 INTRODUCTION

Mating systems are influenced by the spatiotemporal distribution of individuals, which impacts encounter rates and the timing of mating opportunities (Westneat & Stewart, 2003). While social bonds between mates are widespread in birds (Cockburn, 2006), there is now pervasive evidence of mating outside the social bond in 76% of sampled socially monogamous species with biparental care (Brouwer & Griffith, 2019). However, the literature presents a phylogenetic and geographical bias: only <4% of all avian biodiversity and 47% of passerine families have been sampled (Brouwer & Griffith, 2019). Extra‐pair paternity is an important attribute of avian mating systems, showing great variation among species, populations, and individuals of the same species (Cornwallis et al., 2010; Griffith et al., 2002; Petrie & Kempenaers, 1998). Several adaptive and non‐adaptive hypotheses have been suggested to explain this behavior (see Brouwer & Griffith, 2019 for an overview). Briefly, females might engage in extra‐pair copulations to ensure the fertilization of their clutch in case of infertility of their social mate, maximize the genetic diversity among their offspring, obtain “good genes” for their offspring, maximize the genetic compatibility with the genetic mate, seek direct benefits and resources, or avoid male harassment (Birkhead & Møller, 1992; Burke et al., 1989; Colwell & Oring, 1989; Foerster et al., 2003; Hamilton, 1990; Møller, 1988; Sheldon, 1994; Westneat et al., 1990; Wetton & Parkin, 1991; Wolf, 1975). In addition, ecological drivers such as breeding density, breeding synchrony, latitude, life‐history traits and predation rates have been identified to influence extra‐pair paternity rates and suggested to explain variation among and within species (Charmantier & Perret, 2004; Stutchbury & Morton, 1995; Westneat et al., 1990; Wink & Dyrcz, 1999; Yuta & Koizumi, 2016).

Extra‐pair paternity in secondary cavity nesters has been widely investigated in studies that involve breeding in artificial cavities (i.e., nestboxes) (Dunn & Robertson, 1993; Gowaty & Bridges, 1991; Kempenaers et al., 1992). In general, nestboxes have become the standard reference in ecology and evolution because of the convenience of sampling and experimental manipulation (Lambrechts et al., 2010; Wesołowski, 2011) and, therefore, knowledge stemming from these studies is perceived as the reference state. However, nestboxes might represent a source of bias for studies investigating cavity nesters, whose original nesting site is a tree hollow. Tree hollows are cavities naturally occurring in old‐growth stands or excavated by primary cavity nesters, such as woodpeckers. A handful of studies contrasted reproductive performance between birds breeding in natural cavities and nestboxes at the same location and uncovered biological and ecological differences between the two types of cavities (Czeszczewik, 2004; East & Perrins, 1988; Johnson & Kermott, 1994; Llambías & Fernández, 2009; Miller, 2002; Robertson & Rendell, 1990; Sudyka, Di Lecce, Wojas, et al., 2022). Nestboxes differ from tree hollows in several aspects. They are made of plywood or woodcrete (a mixture of timber and concrete) and have standard dimensions and wall thickness. Compared with natural cavities, they provide a far less stable microclimate in terms of temperature and humidity and they have lower buffering capabilities against ambient conditions (Maziarz et al., 2017; Sudyka, Di Lecce, & Szulkin, 2022). However, nestboxes provide protection from nest soaking or flooding, which is very common in natural cavities (Sudyka, Di Lecce, Wojas, et al., 2022; Wesołowski et al., 2002). Moreover, nestboxes and natural cavities differ in terms of ectoparasite loads (Wesołowski & Stańska, 2001), with high levels observed in wooden nestboxes. Predation pressures have been shown to differ between natural cavities and nestboxes, the latter typically providing better protection from a variety of nest predators (Czeszczewik, 2004; Miller, 2002), possibly leading to increased productivity and nesting success (Norris et al., 2018 but see Johnson & Kermott, 1994). There is also evidence that nestboxes may create artificial breeding densities (Perrins, 1979; Tiainen et al., 1984) and increase proximity among individuals, with consequences for reproductive outputs (Pöysä & Pöysä, 2002). Higher breeding density may also increase encounter rates and mating opportunities outside the social bond since most extra‐pair matings occur between close neighbors (Canal et al., 2012; Mayer & Pasinelli, 2013; Møller, 1991; Schlicht et al., 2015). For instance, manipulating nestbox availability increased the likelihood of extra‐pair paternity in some species (Charmantier & Perret, 2004; Gowaty & Bridges, 1991; Stewart et al., 2010), despite the fact that studies in other species failed to find any relationship between breeding density and frequency of extra‐pair paternity (Dunn et al., 1994; Tarof et al., 1998).

Since extra‐pair paternity is the result of females and males interacting in space and time, both the spatial distribution of individuals (i.e., breeding density) and the temporal concentration of fertile females (i.e., breeding synchrony) create opportunities for extra‐pair copulations. Contrasting hypotheses have been suggested regarding breeding synchrony, thereby synchronous or asynchronous breeding might favor extra‐pair matings (see for example Arlt et al., 2004; Stutchbury & Morton, 1995). Under synchronous conditions, females can compare the quality of many males simultaneously (Kempenaers et al., 1992) or alternatively males have the opportunity to seek extra‐pair copulations with many fertile females (Stutchbury & Morton, 1995). In contrast, asynchronous breeding might allow males to seek extra‐pair copulations when their own mate is no longer fertile, once they are free from mate‐guarding (Neudorf, 2004). Artificial breeding densities created by nestboxes might, therefore, interact with breeding synchrony to affect extra‐pair paternity.

Given the extent to which nestboxes differ from natural cavities, criticism has been raised over the ecological validity of results derived from nestbox studies (Lambrechts et al., 2010; Møller, 1992; Wesołowski, 2007, 2011; but see Koenig et al., 1992). It is, therefore, important to establish whether trait variation observed in nestboxes is representative of the natural variation occurring in tree hollows and assess the general significance of the conclusions drawn from nestbox studies. This is true when aiming to quantify the baseline promiscuity levels in both types of cavities and when inferring possible consequences generated by variation in promiscuity in terms of offspring body condition, physiology, survival, or lifetime reproductive success. In fact, these fitness‐related traits have been shown to vary between extra‐pair and within‐pair offspring in some species (Bowers et al., 2015; Foerster et al., 2003; Magrath et al., 2009; Sardell et al., 2012; Schmoll et al., 2009). Here, we investigated variation in extra‐pair paternity levels in blue tits (*Cyanistes caeruleus*) and great tits (*Parus major*) breeding in natural cavities and nestboxes in the same urban forest in Warsaw, Poland. Blue tits and great tits are small passerine cavity nesters, breeding readily in nestboxes and equally widespread in natural and urban environments. They are socially monogamous with biparental care and varying levels of extra‐pair paternity among populations (Cramp & Perrins, 1993; Gullberg et al., 1992; see Brouwer & Griffith, 2019 for an overview of rates of extra‐pair paternity). In this study, we tested whether providing nestboxes influenced the spatial and temporal component of extra‐pair mating behavior by contrasting rates of extra‐pair paternity between two environmentally homogenous plots within the same urban forest: one plot had natural cavities without any nestboxes, while the other plot was supplemented with nestboxes. We predicted that in both species the nestbox plot would have a higher local density of breeding pairs relative to the natural cavity plot. We further predicted that this would translate into higher levels of extra‐pair paternity, specifically a larger number of broods with extra‐pair offspring in the nestbox plot and a higher proportion of extra‐pair offspring in nestboxes.

## 2 MATERIALS AND METHODS

### 2.1 Study site and field methods

Detailed information on the study site and field methods is given in Sudyka, Di Lecce, Wojas, et al., 2022. In short, the study was carried out on wild blue tits and great tits breeding in natural cavities and nestboxes in Bielany Forest, a natural reserve within the city limits of Warsaw (52°17′37.0″ N 20°57′22.6″ E) over two consecutive seasons (2018 and 2019). Bielany Forest (ca. 150 ha) is a remnant of the Mazovian Primeval forest, with multispecies and uneven‐aged stands, and protected under the Natura 2000 scheme. Naturally occurring and excavated cavities were monitored in the SE part of the reserve in a 50 ha area plot, where study birds mainly inhabit cavities in hornbeams (*Carpinus betulus*) and common oaks (*Quercus robur*). In late January 2018, 65 woodcrete Schwegler 1b nestboxes, with a 32 mm diameter entrance, were set up 50 m apart from each other in the NW part of the forest, in an overall area of 15 ha. A distance of 50 m between nestboxes was chosen to match the grid established in other study sites in Warsaw (Corsini et al., 2021) following Krebs (1971), who reported an average nearest neighbur distance of ca. 50 m. Nestboxes were hanged at approximately 2.91 m height with a random orientation, without any protective device against predation (Sudyka, Di Lecce, Wojas, et al., 2022). The minimum distance between the edges of the two plots was 200 m. The two plots share the same environment and are functionally homogeneous: food availability, assessed in the same study years as frass fall collection (Wesołowski & Rowiński, 2014) is uniform between plots, as well as ambient temperature, humidity and sound and air pollution, measured as PM 2.5 concentration (Sudyka, Di Lecce, & Szulkin, 2022; Sudyka, Di Lecce, Wojas, et al., 2022). Moreover, the risk of predation is unlikely to vary at this scale: for instance, in case of martens a distance of 200 m is a territory of one individual/pair (Zalewski & Jędrzejewski, 2006). At the start of the field season, old nests were not present in either of the two cavity types, as nest material naturally degrades in natural cavities (Sudyka, Di Lecce, Wojas, et al., 2022; Wesołowski, 2000), and nestboxes were cleaned to remove old nesting material and/or winter roosts. From the end of March/beginning of April, nest searches started in the natural cavity plot to locate as many active nests as possible at the nest building stage. After 2–3 weeks, search efforts were reduced and individual nest monitoring followed. In May, nest searches were temporarily resumed in order to locate nests of parents that failed their first clutch. In 2019 we also performed a nest search in the nestbox plot, during which 16 nests in natural cavities were discovered. Due to their limited accessibility (4 nests were located in very high or dry branches, and 1 nest was too deep to reach nestlings), and to the high number of nest failures before the nests were accessed (7 nests), we sampled nestlings in only three of these nests (a fourth nest was found close to fledging and thus excluded). Therefore, neither parental nor nestling genetic data from these three nests were included in the main analyses because the low sample size would not allow us to make a rigorous comparison between natural cavities and nestboxes within the plot (see details in Sudyka, Di Lecce, Wojas, et al., 2022). Whenever possible, each cavity was accessed with ladders or by climbing on trees (except for ground‐level cavities) and inspected using portable led lights, mirrors and an NTS200 Digital Inspection Camera (NovoTech Industries Ltd.), equipped with a 8.2 mm camera head at the end of a 5 m long probe. Nestboxes were inspected weekly from the beginning of April to record all breeding events. From the onset of incubation onwards, each nest was monitored individually. Laying date, clutch size, number of hatchlings and number of fledglings were recorded for each nest. During ringing, blood samples were collected from adults and nestlings by puncturing the brachial vein with a sterile needle into heparin‐free capillary tubes and preserved in 99% ethanol at +4°C until DNA isolation (when blood sampling was unsuccessful, a tail feather was collected to ensure that genetic material from all offspring was available for analyses). Blood and tissue (feather or muscle) samples were also collected from dead nestlings and preserved in the same way. We sampled nestlings at the age of 14 days (hatching day = 0) to make sampling time uniform between cavity types, as in natural cavities it is not possible to extract nestlings safely from the nest at a younger age (see Supplementary, Appedix A for information on the proportion of the brood sampled between cavity types). Nestlings were gently pulled out from natural cavities with a “lasso” made out of a metallic wire covered in soft plastic and placed around the neck/body of each nestling. This technique has been developed and used by researchers monitoring cavity‐nesting birds in Białowieża Primeval Forest for several decades (Maziarz et al., 2016; Wesołowski, 2015, 2023). Only first clutches were included in the analyses, as second clutches (i.e., a second nesting attempt after successfully fledging young in an earlier nesting attempt) only occurred five times out of a total of 282 nests across 2 years (1.8%). Nests were marked for coordinates with a GPSMAP® 64 (Garmin).

### 2.2 Genetic analysis, parentage, and sex assignment

**FIGURE 1:**
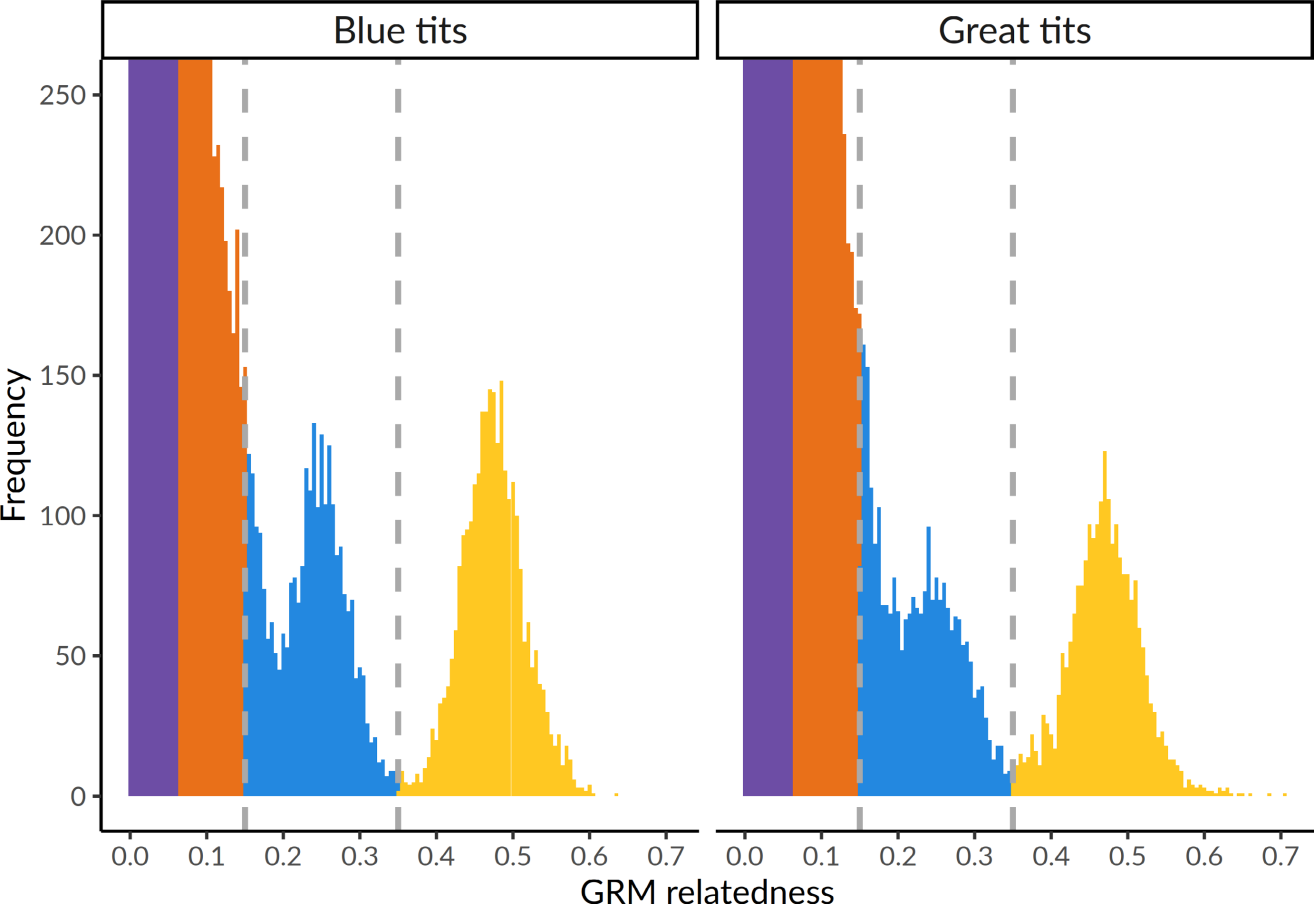
Distribution of GRM values zoomed in on related individuals. Color intervals are indicative of different coefficients of relatedness: purple indicates 1/16; orange 1/8; blue 1/4 (reflecting half‐sibling relationships); and yellow 1/2 (full sibling and parent‐offspring relationships). Vertical dotted lines indicate cut‐off values used to identify half‐siblings, full‐siblings, and extra‐pair paternities.

**FIGURE 2:**
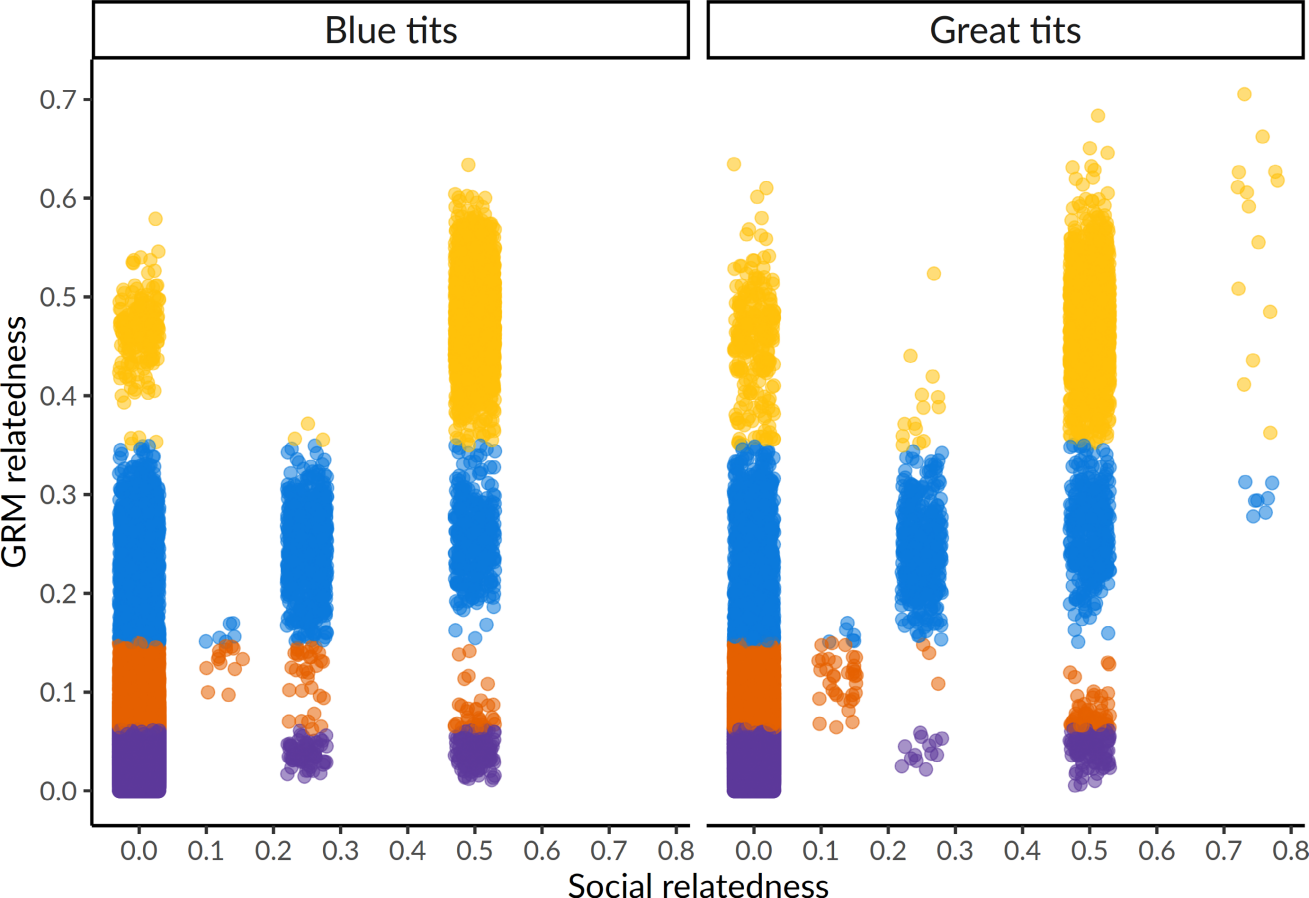
Biplot of GRM and social relatedness values among all individuals illustrating the concordances and discrepancies between the two. GRM and social information are not always concordant and GRM gives in‐depth information on relatedness that is often not represented by social pedigrees. Color intervals are indicative of different coefficients of relatedness: purple indicates 1/16; orange 1/8; blue 1/4 (reflecting half‐sibling relationships); and yellow 1/2 (reflecting full sibling and parent‐offspring relationships).

Genomic DNA was extracted using the Blood Mini kit from 1112 blood samples and the Genomic Mini kit from 71 feather and tissue samples (A&A Biotechnology, Gdynia, Poland) according to the manufacturer's protocol, with the modification of overnight incubation at 37°C. DNA concentration and purity was assessed with a DeNovix DS‐11 spectrophotometer. DNA sequencing was outsourced to Diversity Arrays Technology Pty, Ltd (Canberra, AU) and performed using DArTseqLD, a high‐throughput genotyping by sequencing method that employs genomic complexity reduction using restriction enzyme pairs (Kilian et al., 2012). Details on the DArT sequencing technology can be found in Supplementary, Appendix B. All subsequent analyses were performed in R (version 4.1.2) (R Core Team, 2021) and run separately for each species. We filtered out individuals and loci with call rate below 70% using *dartR* (version 1.9.9.1) (Gruber et al., 2018). Genetic relationships among pooled individuals from natural cavity and nestbox plots were estimated using the function *snpgdsGRM* with the method GCTA (Yang et al., 2011) implemented in *SNPRelate* (version 1.26.0) (Zheng et al., 2012) and represented with a histogram (Figure 1 shows the distribution of relatedness zoomed in on related individuals). The resulting Genomewide Relatedness Matrix (GRM) was compared with a social pedigree of all individuals ringed in the field, created using *ggroups* (version 2.1.0) (Nilforooshan & Saavedra‐Jiménez, 2020). Aligning the GRM against the social pedigree (Figure 2) allowed to detect cases of erroneous pedigree relationships (due to observational errors or brood parasitism) and extra‐pair paternity. Cuckolded fathers, extra‐pair fathers, extra‐pair offspring and broods with unknown parents containing half‐siblings were identified based on discrepancies between the GRM and the social pedigree following Perrier et al. (2018), as here described. Father–offspring pairs (social relatedness = 0.5) showing GRM relatedness estimates above 0.35 were classified as within‐pair paternity (falling within the yellow band in Figure 2). Father–offspring pairs (social relatedness = 0.5) showing GRM relatedness estimates below 0.15 were classified as instances of extra‐pair paternity (falling within the orange/purple band in Figure 2). Adult males with GRM relatedness estimates above 0.35 with offspring from other nests (social relatedness = 0) were identified as extra‐pair fathers (falling within the yellow band in Figure 2). False positives between extra‐pair father and offspring relationships (namely male full siblings from previous years) were identified by checking against field records. It was not possible to identify which particular nestlings were extra‐pair in nests where the social father was not sampled, but we established whether the brood contained full or half‐siblings. Pairs of siblings within a given nest (social relatedness = 0.5) with GRM estimates between 0.15 and 0.35 were classified as half‐siblings (falling within the blue band in Figure 2) and above 0.35 as full siblings (falling within the yellow band in Figure 2). Nestlings with GRM relatedness estimates below 0.1 to both social parents and social siblings (social relatedness = 0.5) were classified as instances of brood parasitism (falling within the orange/purple band in Figure 2). Sex in nestlings was determined using a machine‐learning population assignment approach. First, we identified SNPs that diverged between known females and males by looking for markers with sex differences in heterozygosity and showing high FST and by using BayeScan 2.1 (Brelsford et al., 2017; Foll & Gaggiotti, 2008; Trenkel et al., 2020). BayeScan was run with default parameter options and outlier SNPs were identified with a q‐value below 0.05. The identified markers (11 in blue tits and 7 in great tits) were then used to assign sex to 899 nestlings with *assignPOP* (version 1.2.2) (Chen et al., 2018). Figure S1 shows the assignment accuracy of Monte‐Carlo cross‐validation.

### 2.3 Spatiotemporal parameters

To study the effects of breeding density and breeding synchrony in natural cavities and nestboxes at the local level, for each given nest we estimated: (i) nearest neighbor distance (distance in meters to the nearest conspecific occupied nest); (ii) neighbor density (number of conspecific nests within three different radii around each nest); and (iii) synchronous neighbor density (number of conspecific neighboring females whose fertile period overlapped with that of the focal female, within each of the three chosen radii; the fertile period was calculated from 2 days prior of onset of laying for blue tits and from 5 days for great tits until the penultimate day of laying; Johnson, 2002; Møller, 1991; Yezerinac & Weatherhead, 1997). The three radii used to estimate neighbor density and synchronous neighbor density were chosen as follows. We calculated the median distance between the nest of extra‐pair fathers identified in the population and the nest where they sired extra‐pair offspring, which was equivalent to 96 m in blue tits and 72 m in great tits. As extra‐pair fathers were identified in only a limited number of nests (*n* = 8 blue tit and *n* = 5 great tit nests), we additionally repeated the analysis with radii two and three times larger than the original estimate to identify the most informative distance explaining variation in promiscuity: 192 and 288 m in blue tits and 144 and 216 m in great tits. Our reasoning was that nearest neighbor distance represents the distance to the nearest possible extra‐pair partner, while the other two measures within each radius reflect the number of extra‐pair mating opportunities within the neighborhood of a focal nest. Results for the smallest radius are presented in the main text, while those for the other two are presented in Supplementary (Tables S6 and S8). Additionally, neighbor density and synchronous neighbor density estimation was run again after including data from natural cavities found in the nestbox plot in 2019 (this did not affect our estimates, see Table S4). All calculations were run for each year and each species separately.

### 2.4 Statistical analysis

We performed several models accounting for variation in extra‐pair paternity at three levels: plot, nest, and individual nestling level. At the plot level, to compare promiscuity between cavity types (natural cavity vs. nestbox), we used chi‐square tests on the proportion of nests with extra‐pair paternity relative to the overall number of nests and on the proportion of extra‐pair offspring relative to the overall number of nestlings. We also performed a chi‐square test to determine whether the proportion of extra‐pair nests and extra‐pair offspring differed between study years. We used Mann–Whitney U‐tests on the three spatiotemporal parameters to test for differences between cavity types (also including natural cavities in the nestbox plot found in 2019) and between years. In addition, we used Mann–Whitney *U*‐tests on the proportion of extra‐pair young per nest in nests with extra‐pair paternity between the natural cavity and nestbox plot and between years. For the analyses at the nest level, we employed generalized linear mixed‐effects models with a binomial error distribution and a logit link function to test for the effects of cavity type (binary response: natural cavity vs. nestbox) and the three spatiotemporal parameters on two response variables: occurrence of extra pair paternity in each nest and proportion of extra‐pair young per nest. Occurrence of extra pair paternity for each brood was coded as 0 (no extra‐pair offspring) or 1 (at least one extra‐pair offspring). The three spatiotemporal parameters showed moderate correlations (Table S2) and were, therefore, separately included as covariates in three models. We included mother identity as random effect and laying date and clutch size as covariates. Because of model convergence failure, when testing the occurrence of extra‐pair paternity in great tits we discarded the random term and used generalized linear models. In order to verify that using fixed‐effects models instead of mixed‐effects models did not bias our conclusions, we repeated the models by discarding mother identity as random term in blue tits and present results in the Supplementary (Table S7). When testing the proportion of extra‐pair offspring we excluded nests where the social father was not sampled, as well as two blue tit nests (out of a total of 25) and three great tits nests (out of a total of 25) where less than 50% of the clutch was sampled. In the models at the nest level, we accounted for year differences in spatiotemporal parameters by z‐scaling these variables. We repeated these models by including year as a fixed factor without z‐scaling numerical predictors and present the results in Supplementary (Tables S9 and S10). Additionally, at the individual level, we applied a generalized linear mixed model with a binomial error distribution and a logit link function to investigate the effect of cavity type and sex on being an extra‐pair offspring. The response variable was offspring status as within‐pair or extra‐pair (0/1). A similar model was used to test for the effect of being extra‐pair on a proxy of fitness (i.e., successful fledging of nestlings sampled at day 14) in the two cavity types (natural cavity vs. nestbox); offspring were coded as 0 (not fledged) or 1 (fledged) as response variable. In both these models, cavity type, year and nestling sex were included as fixed factors, and body index, laying date and clutch size were covariates. Body index was calculated as ‘scaled mass index’ following Peig and Green (2009). An interaction between cavity type and sex was also included. There is evidence that females are able to bias the sex ratio of their offspring (Svensson & Nilsson, 1996) and sex allocation theory predicts that it would be adaptive for females to bias the sex ratio of their extra‐pair offspring towards males (Trivers & Willard, 1973). It is possible that this mechanism might have acted stronger in nestboxes where we expected higher overall extra‐pair paternity levels. In the model for fledging success, offspring status as within‐pair or extra‐pair was also included as fixed factor. Nest identity was introduced as random effect in both models. All models were checked for dispersion, zero inflation and multicollinearity (VIF scores in each model never exceeded 3). All statistical analyses were performed in R 4.1.3 (R Core Team, 2021), separately for each species.

## 3 RESULTS

### 3.1 Genetic paternity

For blue tits, a total of 118 adults and 497 nestlings were successfully sequenced, together with 114 great tit adults and 402 nestlings. Out of these, 62 (53%) adult blue tits, 226 (46%) blue tit nestlings, 74 (65%) adult great tits, and 207 (52%) great tit nestlings were sampled in natural cavities. In each species, the distribution of GRM values among individuals from the two plots combined together (Figure 1) showed the presence of parent‐offspring (yellow), full sibling (yellow), and half‐sibling relationships (blue). Males with 0.5 social relatedness and GRM relatedness below 0.15 with their social offspring represented cuckolded fathers (*n* = 25 in blue tits and *n* = 25 in great tits; Figure 2). Males with 0 social relatedness and GRM relatedness above 0.35 with offspring from other nests were identified as extra‐pair fathers (*n* = 7 blue tits and *n* = 4 great tits; Figure 2). Extra‐pair offspring were identified by having 0.5 social relatedness and GRM relatedness below 0.1 with their social father and 0 social relatedness and GRM relatedness above 0.35 with males from other nest (*n* = 44 in blue tits and *n* = 50 in great tits; Figure 2). In nests with unknown father identity, nestlings with 0.5 social relatedness and GRM relatedness between 0.15 and 0.35 with each other were identified as half‐siblings (*n* = 3 nests in blue tits and *n* = 3 nests in great tits; Figure 2). One blue tit nestling with GRM relatedness estimates below 0.1 to the other nestlings in the brood and to both the social parents (0.5 social relatedness) was classified as an instance of brood parasitism (Figure 2).

### 3.2 No clear effect of cavity type on extra‐pair paternity at the plot level

**FIGURE 3:**
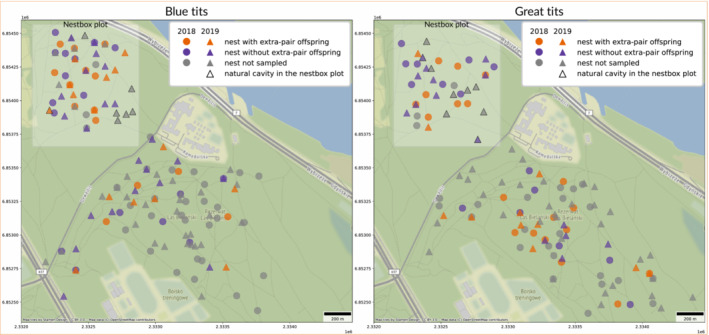
Map of blue tit and great tit nests from Bielany Forest in 2018 and 2019 with information on extra‐pair paternity. Gray symbols represent clutches that failed before day 14 and inaccessible nests in natural cavities.

**FIGURE 4:**
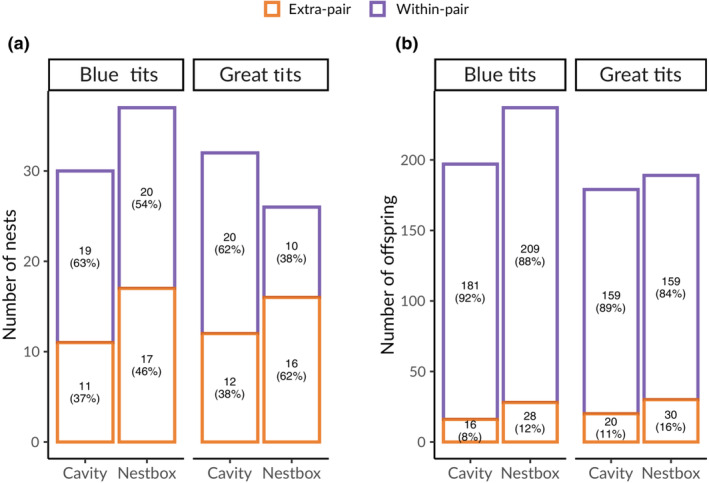
Number of extra‐pair and within‐pair (a) nests and (b) offspring in blue tits and great tits by cavity type (percentages in parenthesis).

**FIGURE 5:**
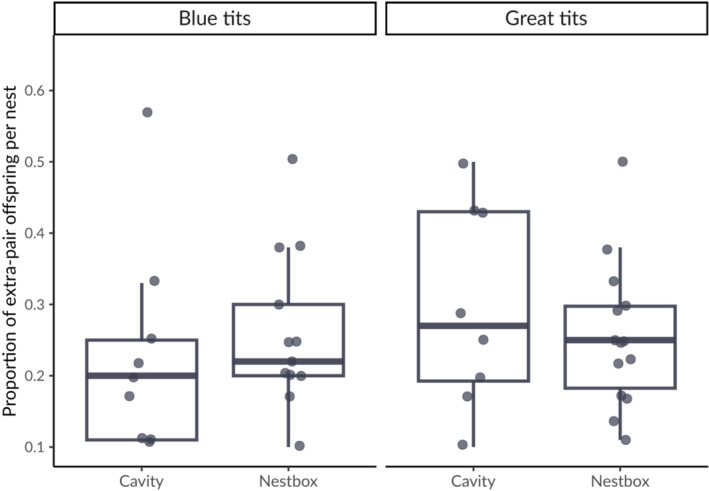
Distribution of the proportion of extra‐pair offspring relative to within‐pair offspring per nest in blue tits and great tits, by cavity type. Nests where social father was sampled were included.

Over 2 years, in blue tits we recorded 87 nesting attempts in natural cavities and 46 in nestboxes. Out of the 87 natural cavities, 32 were inaccessible because they were located in very high and/or dry branches, to which climbing was not possible for safety reasons, or because the nest was too deep to reach and extract nestlings; we obtained information on extra‐pair paternity for 30 broods. In great tits, we recorded 88 nesting attempts in natural cavities (of which 18 were inaccessible; in 32 we were able to obtain extra‐pair paternity information) and 29 in nestboxes. Nest searches proved to be highly effective, because most nests were found at building also in natural cavities (81% nests in 2018 and 80% in 2019; Sudyka, Di Lecce, Wojas, et al., 2022). Figure 3 shows the distribution of nests within Bielany Forest, with information on extra‐pair paternity. In blue tits, 37% of broods in the natural cavity plot (*n* = 11 out of 30) and 46% in the nestbox plot (*n* = 17 out of 37) contained at least one extra‐pair offspring. 8% of blue tit nestlings in the natural cavity plot (*n* = 16 out of 197) and 12% in the nestbox plot (*n* = 28 out of 237) were extra‐pair offspring (Figure 4). In great tits, 38% of broods in the natural cavity plot (*n* = 12 out of 32) and 62% in the nestbox plot (*n* = 16 out of 26) contained at least one extra‐pair offspring. 11% of great tit nestlings in the natural cavity plot (*n* = 20 out of 179) and 16% in the nestbox plot (*n* = 30 out of 189) were sired by males other than their social father (Figure 4). Despite recording both a larger number of broods with extra‐pair offspring and more extra‐pair offspring in nestboxes in both species, the trend was not statistically supported, either in terms of proportion of extra‐pair broods (blue tits: χ^2^ = 0.267, df = 1, *p* = .605; great tits: χ^2^ = 2.427, df = 1, *p* = .119) or in terms of proportion of extra‐pair offspring (blue tits: χ^2^ = 1.230, df = 1, *p* = .267; great tits: χ^2^ = 1.352, df = 1, *p* = .245). Differences between years in the proportion of broods containing at least one extra‐pair offspring lacked statistical support (blue tits: χ^2^ = 0.073, df = 1, *p* = .787, *n* = 67; great tits: χ^2^ = 0.238, df = 1, *p* = .626, *n* = 58), similarly to differences between years in the proportion of extra‐pair offspring in the population (blue tits: χ^2^ = 0.319, df = 1, *p* = .572, *n* = 434; great tits: χ^2^ = 2.801, df = 1, *p* = .094, *n* = 368). No brood consisted entirely of extra‐pair young either in blue tits or great tits over the 2 years in either plot. Table S3 shows the number of broods with number of extra‐pair offspring per cavity type. There was no statistical support for a difference in the proportion of extra‐pair young per nest between natural cavity and nestbox plots in nests with extra‐pair offspring, neither in blue tits (W = 71.5, *p* = .867, *n* = 23) nor in great tits (W = 90.5, *p* = .403, *n* = 22) (Figure 5). Differences in the proportion of extra‐pair offspring between study years also lacked statistical support (blue tits: W = 74, *p* = .596; great tits: W = 44, *p* = .347).

### 3.3 Spatiotemporal parameters differed between cavity types in blue tits but not in great tits

In blue tits, the average breeding density across the two study years was 11.8 pairs/10 ha in the natural cavity plot and 17.7 pairs/10 ha in the nestbox plot, while in great tits there were 10 and 12.2 breeding pairs in the natural cavity and nestbox plot, respectively. At the plot level, there was statistical support for a longer nearest neighbor distance in the natural cavity plot than in the nestbox plot in blue tits but not in great tits (despite a tendency for longer nearest neighbor distance in nestboxes; Table 1). In blue tits, neighbor density was lower in the natural cavity plot than in the nestbox plot within a radius of 96 and 192 m and synchronous neighbor density was lower in all three radii in the natural cavity plot (Table 1). In great tits, neighbor density within 216 m was higher in the natural cavity plot than in the nestbox plot and synchronous neighbor density within 144 m was lower in the natural cavity plot than in the nestbox plot (Table 1). The same trends were statistically supported in blue tits when including natural cavities found in the nestbox plot in 2019 (Table S4). In great tits no differences between cavity types were observed when including natural cavities found in the nestbox plot (Table S4). Differences in spatiotemporal parameters between the two study years were not statistically supported in either species (Table S5).

**TABLE 1:** Mann–Whitney *U*‐tests on nearest neighbor distance, neighbor density and synchronous neighbor density in the three investigated radii for each species.

	Nearest neighbor distance				Radius (m)	Neighbor density				Synchronous neighbor density			
	W	*p*	Median in the cavity plot (m)	Median in the nestbox plot (m)		W	*p*	Median number in the cavity plot (range)	Median number in the nestbox plot (range)	W	*p*	Median number in the cavity plot (range)	Median number in the nestbox plot (range)
Blue tits (*n* = 131)	2424	**.024**	58	48	**96**	1394	**.006**	2 (0–7)	3 (1–6)	1224	**<.001**	1 (0–5)	2 (0–6)
					**192**	1396.5	**.007**	9 (1–17)	11 (5–18)	1290	**.001**	4 (0–13)	6.5 (0–13)
					**288**	1911	.834	17 (2–31)	17.5 (11–22)	1538	**.044**	9 (1–19)	12 (1–18)
Great tits (*n* = 116)	1024	*.131*	50	54	**72**	1235	.861	1 (0–4)	1 (0–3)	1079.5	.207	0 (0–3)	1 (0–2)
					**144**	1393.5	.398	4 (0–11)	4 (1–8)	888.5	**.016**	2 (0–7)	3 (0–7)
					**216**	1572.5	**.047**	9 (2–21)	7 (1–15)	980.5	*.072*	5 (0–12)	6 (0–13)

*Note*: Significant differences (*p* < .05) are in bold, and trends (*p* < .2) are in italics.

### 3.4 Relationship between spatiotemporal parameters and extra‐pair paternity

**FIGURE 6:**
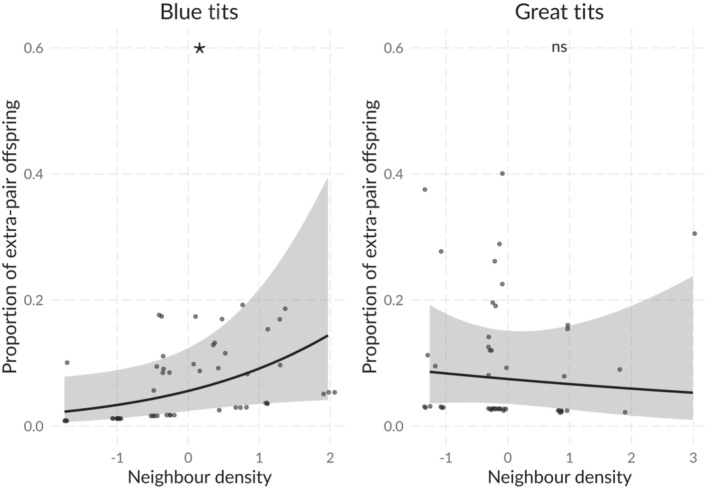
Proportion of extra‐pair offspring per nest (number of extra‐pair offspring over total number of nestlings in the nest) in relation to the number of neighbors within 96 m in blue tits and 72 m in great tits (based on values reported in Table 3). Predicted values with 95% confidence interval are shown. ns, non significant; * denotes .01 ≤ *p* ≤ .05.

At the nest level, there was no statistical support for an effect of nearest neighbor distance on the occurrence of extra‐pair paternity in blue tits or great tits (Table 2). Similarly, no effect of neighbor density and synchronous neighbor density on the occurrence of extra‐pair paternity was detected either in blue tits or in great tits in any of the three investigated radii (Table 2; Table S6). In models for great tits the likelihood of having extra‐pair offspring at the nest was higher in nests with later laying date (Table 2; Table S6). Fixed‐effects models repeated on blue tit data (Table S7) showed similar results to the mixed‐effects models (Table 2; Table S6), suggesting that discarding the random effect in models for great tits did not bias our conclusions. In blue tits, we found statistical support for a higher proportion of extra‐pair offspring per nest with increasing neighbor density within 96 m (Table 3; Figure 6), but no effect was apparent within 192 and 288 m (Table S8). Moreover, we detected no effect of nearest neighbor distance and synchronous neighbor density in any of the three investigated radii (Table 3; Table S8). In great tits, there was no evidence for an effect of the investigated spatiotemporal parameters on the proportion of extra‐pair offspring per nest (Table 3; Table S8). Mixed‐effects models including year provided support for the same associations as the simplified models (with z‐scaling of numerical parameters) in both species (Table S9; Table S10).

**TABLE 2:** Generalized linear mixed models for blue tits and generalized linear models for great tits with the presence of extra‐pair paternity in the nest (0/1) as the dependent variable.

Occurrence of extra‐pair paternity in the nest						

Blue tits (*n* = 52)	Estimate (SE)	Pr (>χ^2^)	Great tits (*n* = 48)	Estimate (SE)	Pr (>χ^2^)
Model a	Nearest neighbor distance	−0.332 (0.344)	0.335	Nearest neighbor distance	−0.030 (0.357)	0.932
	Cavity type	0.275 (0.658)	0.676	Cavity type	0.901 (0.697)	*0.191*
	Laying date	0.067 (0.365)	0.856	Laying date	1.082 (0.465)	**0.008**
	Clutch size	−0.047 (0.193)	0.806	Clutch size	−0.352 (0.236)	*0.121*
Model b	Neighbor density	0.418 (0.359)	0.245	Neighbor density	0.606 (0.412)	*0.122*
	Cavity type	0.313 (0.639)	0.624	Cavity type	1.053 (0.724)	*0.139*
	Laying date	−0.008 (0.385)	0.984	Laying date	1.191 (0.494)	**0.005**
	Clutch size	−0.083 (0.201)	0.679	Clutch size	−0.357 (0.239)	*0.117*
Model c	Synchronous neighbor density	0.050 (0.383)	0.896	Synchronous neighbor density	0.545 (0.388)	*0.151*
	Cavity type	0.496 (0.717)	0.489	Cavity type	0.658 (0.722)	0.363
	Laying date	0.148 (0.365)	0.685	Laying date	1.217 (0.500)	**0.005**
	Clutch size	−0.023 (0.195)	0.905	Clutch size	−0.302 (0.234)	*0.179*

*Note*: Cavity type, laying date, clutch size, nearest neighbor distance (model a), neighbor density (model b), and synchronous neighbor density (model c) within 96 m in blue tits and 72 m in great tits were included as predictors. Social mother identity was introduced as random effect in models for blue tits. Reference levels were: natural cavity (cavity type) and 2018 (year). Significant differences (*p* < .05) are in bold, and trends (*p* < .2) are in italics.

**TABLE 3:** Generalized linear mixed models with proportion of extra‐pair offspring per nest as the dependent variable.

Proportion of extra‐pair offspring per nest						

Blue tits (*n* = 50)	Estimate (SE)	Pr (>χ^2^)	Great tits (*n* = 44)	Estimate (SE)	Pr (>χ^2^)
Model a	Nearest neighbor distance	−0.425 (0.292)	*0.145*	Nearest neighbor distance	0.262 (0.260)	0.313
	Cavity type	0.124 (0.540)	0.818	Cavity type	0.204 (0.495)	0.681
	Laying date	−0.007 (0.314)	0.983	Laying date	0.315 (0.247)	0.201
	Clutch size	−0.181 (0.156)	0.248	Clutch size	−0.150 (0.158)	0.342
Model b	Neighbor density	0.565 (0.271)	**0.037**	Neighbor density	0.045 (0.250)	0.858
	Cavity type	0.095 (0.517)	0.854	Cavity type	0.265 (0.476)	0.578
	Laying date	−0.067 (0.304)	0.827	Laying date	0.337 (0.242)	*0.163*
	Clutch size	−0.219 (0.152)	*0.151*	Clutch size	−0.147 (0.151)	0.332
Model c	Synchronous neighbor density	0.057 (0.328)	0.862	Synchronous neighbor density	0.116 (0.231)	0.615
	Cavity type	0.400 (0.625)	0.522	Cavity type	0.183 (0.492)	0.710
	Laying date	0.079 (0.311)	0.799	Laying date	0.354 (0.239)	*0.138*
	Clutch size	−0.164 (0.163)	0.313	Clutch size	−0.139 (0.148)	0.348

*Note*: Cavity type, laying date, clutch size, nearest neighbor distance (model a), neighbor density (model b), and synchronous neighbor density (model c) within 96 m in blue tits and 72 m in great tits were included as predictors. Mother identity was introduced as random effect. Reference levels were: natural cavity (cavity type) and 2018 (year). Significant differences (*p* < .05) are in bold, and trends (*p* < .2) are in italics.

### 3.5 Extra‐pair paternity and cavity types at the individual level

**FIGURE 7:**
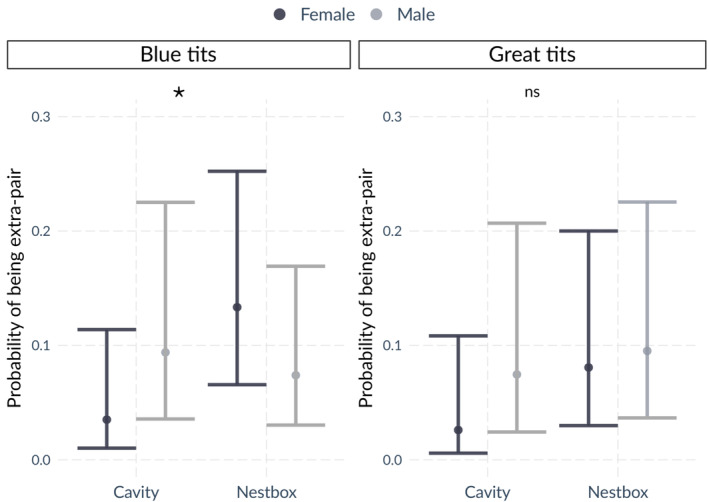
Probability of being extra‐pair for blue tit and great tit offspring with respect to cavity type and sex. Predicted values with 95% confidence interval are shown. ns, non significant; * denotes .01 ≤ *p* ≤ .05.

At the individual level, there was statistical support for an effect of cavity type on the sex‐specific probability of being an extra‐pair blue tit. Thus, female nestlings had higher probability of being extra‐pair in the nestbox plot than in the natural cavity plot (Table 4; Figure 7). In great tits, no effect of the investigated variables was detected on the probability of being extra‐pair (Table 4). In blue tits, the mixed‐effects model provided no support for a difference in late mortality rate (between day 14 and before fledging the nest) between extra‐pair and within‐pair young (χ^2^ = 0.041, *p* = .841) or between sexes (χ^2^ = 1.637, *p* = .201). In great tits, low sample size led to model convergence issues, precluding testing for successful fledging.

**TABLE 4:** Generalized linear mixed model testing the probability of nestlings of being within‐pair or extra‐pair (0/1; dependent variable), with cavity type, year, nestling sex, laying date, clutch size, and body index as predictors.

Probability of being extra‐pair				
	Blue tits (*n* = 401)		Great tits (*n* = 333)	
	Estimate (SE)	Pr (>χ^2^)	Estimate (SE)	Pr (>χ^2^)
Cavity type	1.442 (0.658)	0.302	0.617 (0.551)	0.262
Year	−0.323 (0.500)	0.516	0.842 (0.582)	*0.148*
Clutch size	−0.109 (0.147)	0.456	−0.030 (0.184)	0.870
Laying date	0.028 (0.268)	0.918	0.266 (0.259)	0.304
Sex	1.046 (0.647)	0.858	0.489 (0.373)	*0.190*
Body index	−0.387 (0.216)	*0.074*	−0.038 (0.230)	0.870
Cavity type × Sex	−1.703 (0.795)	**0.032**		

*Note*: Reference levels were: natural cavity (cavity type), 2018 (year), and female (sex). The non‐significant interaction between cavity type and sex was removed from the final model in great tits. Significant differences (*p* < .05) are in bold, and trends (*p* < .2) are in italics.

## 4 DISCUSSION

We found no differences in levels of extra‐pair paternity at the plot level in blue tits and great tits breeding in natural cavities and nestboxes within the same urban forest. The analysis of blue tit data indicated that nearest neighbor distance was longer, and neighbor density and synchronous neighbor density were lower in natural cavities compared with nestboxes (but note that differences in breeding density are small; Table 1). We found statistical support for a positive relationship between the proportion of extra‐pair offspring in the nest and neighbor density within 96 m in blue tits. In great tits no apparent effects of cavity type or spatiotemporal parameters were detected. Despite the altered spatiotemporal distribution of individuals observed in the nestbox plot in blue tits, our results do not provide evidence that nestboxes per se affect variation in extra‐pair paternity.

### 4.1 Extra‐pair paternity and cavity type

Rates of extra‐pair paternity for blue tits and great tits breeding in nestboxes were similar to those observed in natural cavities. In both species, no difference between cavity types emerged in terms of proportion of mixed broods, the overall proportion of extra‐pair offspring, or proportion of extra‐pair offspring per nest. This contrasts with our predictions of higher extra‐pair paternity levels in the nestbox plot. To date, several studies have compared and uncovered differences in breeding phenology and reproductive success between birds breeding in natural cavities and nestboxes. However, very few studies have investigated whether mating systems might be affected by cavity type (Barber et al., 1996; Kaluthota & Rendall, 2017; Llambías & Fernández, 2009). Our results confirm previous observations of similar extra‐pair paternity levels in tree swallows (*Tachycineta bicolor*) breeding in natural cavities and nestboxes (Barber et al., 1996). On the other hand, they do not match patterns found in western house wrens (*Troglodytes aedon parkmanii*), where nestbox studies reported two to three times higher rates of polygyny in nestboxes compared with natural cavities, suggesting that the distribution of cavities and the ability of males to defend their territories might limit levels of extra‐pair paternity (Kaluthota & Rendall, 2017 and references within). This might indicate a species‐specific pattern, since in this study the type of cavity (natural vs. artificial) did not correlate with extra‐pair paternity in blue tits and great tits (Table 2). Therefore, inference drawn from nestbox studies might adequately represent the natural variation of traits in some species but not in others, and is likely to be mediated by the extent to which nestbox distribution deviates from natural cavity distribution.

We found that extra‐paternity increased as the season progressed independently of cavity type in great tits (Table 2; no difference in laying date between cavity types was observed in our populations; Sudyka, Di Lecce, Wojas, et al., 2022). This is in line with several studies (Beheler & Rhodes Jr., 2003; Lubjuhn et al., 2001; Major & Barber, 2004; but see Stewart et al., 2010). Females might gain extra‐pair copulations more easily later in the season when their mates are caring for fledglings. Alternatively, females that paired later in the season might be in poor‐quality habitats or mated with poor‐quality mates and, therefore, more likely to engage in extra‐pair copulations (Møller, 1992). Higher extra‐pair paternity in early broods has been suggested to occur in migratory species, when mates are not able to accurately assess their partner quality, resulting in hasty pairings with poor‐quality individuals (Spottiswoode & Møller, 2004). However, this explanation does not likely apply to blue tits and great tits from Bielany Forest, which are mostly residents (or short‐space migrants).

At the individual level, we found an association between offspring sex and cavity type in blue tits: extra‐pair offspring were female‐biased in the nestbox plot relative to the natural cavity plot (Figure 7). It is not straightforward to explain this observation, which is opposite to the expected outcome of sex allocation theory predicting that females should produce more sons than daughters among extra‐pair offspring, since male offspring yield greater fitness benefits (Sheldon & Ellegren, 1996; Westneat et al., 1995). It is possible that the observed difference is not related to paternity but to a measure of environmental or parental quality that co‐varies with extra‐pair paternity, which we did not assess. For instance, females in poor condition are predicted to produce more often female than male offspring (Cockburn et al., 2002; Dietrich‐Bischoff et al., 2006). However, we assessed food availability in the same study years as frass fall collection and feather coloration of breeding adults as an indicator of parental condition, and we found no difference between the two cavity types (Janas et al., 2022 preprint; Sudyka, Di Lecce, Wojas, et al., 2022). Alternatively, since blue tits produced fewer fledgings in nestboxes than in natural cavities in the same forest (Sudyka, Di Lecce, Wojas, et al., 2022), this result might stem from early selective mortality of male offspring in nestboxes, as males require a bigger investment than females.

### 4.2 Extra‐pair paternity and spatiotemporal parameters

Overall nest density was comparable between the nestbox and the natural cavity plot. Breeding densities were higher than those observed in a natural habitat such as Białowieża National Park (average 4.0 pairs/10 ha for blue tits and 4.9 pairs/10 ha for great tits; Wesołowski et al., 2010), but comparable with those found in nestbox studies in other urban and forest habitats (Dhondt, 2010). At the local scale, we found evidence for shorter nearest neighbor distance in nestboxes than in natural cavities in blue tits. We also found higher neighbor density and synchronous neigbour breeding density within a radius of 96 and 192 m of the focal nest in nestboxes (although the difference between cavity types was small; Table 1). This result was further corroborated when natural cavities found in the nestbox plot in 2019 were included (Table S4). In great tits differences in neighbor density within 216 m and synchronous neighbor density within 144 m between cavity types were not confirmed when including natural cavities found in the nestbox plot (Table S4). Our results for blue tits seem to be in line with previous observations of nestboxes creating artificially high breeding densities compared with natural populations, but this pattern might not be general across species as it was not apparent in great tits. Furthermore, our data in blue tits suggest that the proportion of extra‐pair offspring per nest increased with neighbor density within 96 m (Table 3). Previous studies have shown a positive effect of breeding density and synchrony on extra‐pair paternity and even a positive interaction term between the two variables (Mayer & Pasinelli, 2013; Stewart et al., 2010; Thusius et al., 2001; Westneat & Mays, 2005). At greater breeding densities, potential extra‐pair mates might be more accessible and searching costs for them might be low (Birkhead & Møller, 1992; Westneat et al., 1990). At the same time, breeding synchrony may facilitate extra‐pair mate choice by females (Stutchbury & Morton, 1995). However, there is also evidence of a negative effect of breeding synchrony on extra‐pair paternity, which may derive from increased risks of paternity loss or male harassment (Dunn et al., 1994; Olsen et al., 2008; Stewart et al., 2006; Westneat & Gray, 1998). This difference among studies and species is likely linked to species‐specific differences pertaining to which sex initiates or controls extra‐pair copulations and differences in mate‐guarding behavior. In our study, the distance to the nearest neighbor was shorter in the nestbox plot than in the natural cavity plot in blue tits. However, the proximity of the nearest neighbor did not affect the occurrence of extra‐pair paternity in either species (Table 2). Nearest neighbors have been identified as sires of extra‐pair offspring in some species, where nearest neighbor distance influences the frequency of extra‐pair paternity (Bollinger & Gavin, 1991; Gray, 1997; Perreault et al., 1997). There is also evidence that extra‐pair sires travel longer distances than the first neighboring nest (Balenger et al., 2009; Meek et al., 1994; Rowe et al., 2001; Westneat & Mays, 2005). These contrasting results have also been found within the same species: for instance, in blue tits Charmantier and Perret (2004) found that nearest neighbor distance influenced extra‐pair paternity, but nearest neighbors accounted only for 39.3% of extra‐pair paternities, confirming previous results showing that extra‐pair sires were not always closest neighbors (Kempenaers et al., 1997). Similarly, six of the seven extra‐pair fathers identified in our population sired offspring farther away than the closest neighboring nest, at 91, 96 (two extra‐pair fathers), 100, 140 and 453 m. Likewise in great tits three out of four identified extra‐pair fathers sired offspring farther away than the closest neighboring nest (two at 72 and one at 81 m). In our population all identified extra‐pair fathers sired nestlings in the same plot (e.g. natural cavity plot or nestbox plot) where they had their social nest.

### 4.3 Conclusions and outlook

Our data provided evidence of altered spatiotemporal parameters in nestboxes compared with natural cavities in blue tits, although quantitative differences in neighbor density and synchronous neighbor density were small (Table 1). This complements previous knowledge of the differences introduced by nestboxes. However, our results offered no evidence for an effect of nestboxes per se on levels of extra‐pair paternity in the population. We acknowledge that there could be a difference in the probability of finding nests between the natural cavity and nestbox plot, because natural cavities have to be actively searched for and nestboxes intrinsically do not. Therefore, we might have missed several nests in the natural cavity plot, despite our extensive search efforts early in the season and in May (see Materials and Methods). However, we do not expect that this might have affected our estimation of spatiotemporal parameters in the natural cavity plot (Table 1). Another potential limitation of the study is that natural cavities and nestboxes were not interspersed in the same plot. This study design was chosen to ensure a random breeding site choice, due to inter‐ and intra‐specific competition and quality‐dependent preference towards a particular cavity type (see Sudyka, Di Lecce, Wojas, et al., 2022 for a more detailed justification of this design). The two plots are located within the same homogenous forest, and food availability, climatic conditions and anthropogenic variables, assessed during the study years, were uniform between them (see Materials and Methods). Therefore, it is unlikely that the variation that we observed might stem from ecological differences between the plots. Importantly however, these limitations are unlikely to influence the key finding of this study – namely that in our study population, levels of extra‐pair paternity do not differ between natural cavities and nestboxes. Given that extra‐pair paternity is the result of multiple ecological factors acting on individual decisions, from which variation at the population level stems, we encourage further comparisons of cavity nesters breeding in natural nesting sites and nestboxes. Additionally, we encourage researchers to follow the recommendations given by Lambrechts et al. (2010) and Wesołowski (2011) to ensure adequate reporting of nestbox setups, in terms of nestbox dimension, their distribution and density, frequency of old material removal, presence of anti‐predator devices, frequency of inspections of nestboxes and characteristics of the study site (all of these variables are likely to have an impact on reproductive success, fitness and possibly variation in levels of extra‐pair paternity). Following these recommendations would promote straightforward ecological comparisons among studies, and allow for an easier assessment of the validity of study conclusions relative to the reference state.

## AUTHOR CONTRIBUTIONS

**Irene Di Lecce:** Conceptualization (equal); data curation (lead); formal analysis (lead); investigation (equal); methodology (lead); writing – original draft (lead); writing – review and editing (equal). **Charles Perrier:** Conceptualization (supporting); formal analysis (supporting); methodology (supporting); writing – review and editing (supporting). **Marta Szulkin:** Conceptualization (equal); formal analysis (supporting); investigation (supporting); methodology (supporting); resources (lead); writing – review and editing (equal). **Joanna Sudyka:** Conceptualization (equal); data curation (supporting); funding acquisition (supporting); investigation (equal); methodology (supporting); writing – review and editing (equal).

## CONFLICT OF INTEREST STATEMENT

We declare none.

## Supporting information


Table S1–S10.

## Data Availability

The data that support the findings of this study are available in FigShare at https://doi.org/10.6084/m9.figshare.21905166.v1.
