# Supplementary material for: Extra‐pair paternity, breeding density, and synchrony in natural cavities versus nestboxes in two passerine birds
Source: Ecol Evol. 2023 Jun 8;13(6):e10163. doi: 10.1002/ece3.10163 (PMC10249044; doi:10.1002/ece3.10163)
Supplement: Supplementary file 1 — Table S1–S10. [file ECE3-13-e10163-s001.docx]

**APPENDIX**

**Appendix A. Proportion of the brood sampled between cavity types**

We tested whether the proportion of sampled nestlings (number of nestlings which we were able to sample over total number of nestlings that hatched successfully in the nest) differed between cavity types (Table S1). There was no difference between cavity types in blue tits but there was a tendency in great tits for a lower proportion of sampled nestlings in natural cavities. After excluding the four natural cavities where we were not able to sample all nestlings in the nest, a weaker tendency persisted (W = 326, P = 0.134), indicating that this result was not due to a differential sampling effort between cavity types. We therefore retained these nests in subsequent analyses. Very few dead chicks were sampled in both natural cavities and nestboxes and in both cases at d14. This is because, usually prior to our visits, parents removed dead chicks small enough to be carried out of the nests. Since we did not sample nestlings at hatching, we were not able to investigate whether this difference among the two cavity types stemmed from differential mortality between extra-pair and within-pair young or between sexes at early nestling stages. The tendency to sample larger proportion of great tit nestlings in nestboxes was effected by a general tendency in great tits to produce more fledglings in nestboxes compared to natural cavities, reported in Sudyka, Di Lecce, Wojas, et al., 2022.

**Table S1 –** Proportion of sampled nestlings per nest between cavity types in the two study species.

|  | **Mean (SD) in natural cavities** | **Mean (SD) in nestboxes** | **Wilcoxon rank sum test** | |  |
| --- | --- | --- | --- | --- | --- |
|  |  |  | **W** | **P** | **n** |
| **Blue tits** | 0.897 (0.140) | 0.806 (0.247) | 244 | 0.295 | 53 |
| **Great tits** | 0.778 (0.227) | 0.875 (0.196) | 371.5 | 0.071 | 48 |

**Appendix B. Genotyping**

DNA sequencing was outsourced to Diversity Arrays Technology Pty, Ltd and performed using DArTseqLD, a high-throughput genotyping by sequencing method that employs genomic complexity reduction using restriction enzyme pairs (Kilian et al., 2012). DArTseq™ represents a combination of DArT complexity reduction methods and next generation sequencing platforms (Sansaloni et al., 2011; Kilian et al., 2012; Courtois et al., 2013; Raman et al., 2014; Cruz et al., 2013). Therefore, DArTseq™ represents a new implementation of sequencing of complexity reduced representations (Altshuler et al., 2000) and more recent applications of this concept on next generation sequencing platforms (Baird et al., 2008; Elshire et al., 2011). Similarly to DArT methods based on array hybridization, the technology is optimized for each organism and application by selecting the most appropriate complexity reduction method (both the size of the representation and the fraction of a genome selected for assays). Based on testing several enzyme combinations for complexity reduction, Diversity Arrays Technology Pty Ltd selected the SbfI-HpaII method for the two species of interest – the great tit *Parus major* and the blue tit *Cyanistes caeruleus*. DNA samples were processed in digestion/ligation reactions principally following Kilian et al. (2012) but replacing a single SbfI-compatible adaptor with two different adaptors corresponding to two different Restriction Enzyme (RE) overhangs. The SbfI-compatible adapter was designed to include Illumina flowcell attachment sequence, sequencing primer sequence and “staggered”, varying length barcode region, similar to the sequence reported by Elshire et al., 2011). Reverse adapter contained flowcell attachment region and HpaII-compatible overhang sequence. Only “mixed fragments” (SbfI-HpaII) were effectively amplified in 30 rounds of PCR using the following reaction conditions:

1. 94̊ C for 1 min

2. 30 cycles of: 94̊ C for 20 sec

58̊ C for 30 sec

72̊ C for 45 sec

3. 72̊ C for 7 min

After PCR equimolar amounts of amplification products from each sample of the 96-well microtiter plate were bulked and applied to c-Bot (Illumina) bridge PCR followed by sequencing on Illumina Hiseq2500. The sequencing (single read) was run for 77 cycles. Sequences generated from each lane were processed using proprietary DArT analytical pipelines. In the primary pipeline, the *fastq* files were first processed to filter away poor quality sequences, applying more stringent selection criteria to the barcode region compared to the rest of the sequence. In that way, the assignments of the sequences to specific samples carried in the “barcode split” step were highly reliable (Kilian et al., 2012).

Filtering was performed on the raw sequences using the following parameters:

| Filter | Filter Parameters |  |  |  |
| --- | --- | --- | --- | --- |
| Barcode region | Min Phred pass score 30, Min pass percentage 75 | | | |
| Whole read | Min Phred pass score 10, Min pass percentage 50 | | | |

Approximately 1,410,000 sequences per sample were identified and used in marker calling. Finally, identical sequences were collapsed into “fastqcoll files”. The fastqcoll files were “groomed” using DArT PL’s proprietary algorithm which corrects low quality base from singleton tag into a correct base using collapsed tags with multiple members as a template. The “groomed” fastqcoll files were used in the secondary pipeline for DArT PL’s proprietary SNP and SilicoDArT (presence/absence of restriction fragments in representation) calling algorithms (DArTsoft14). For SNP calling, all tags from all libraries included in the DArTsoft14 analysis were clustered using DArT PL’s C++ algorithm at the threshold distance of 3, followed by parsing of the clusters into separate SNP loci using a range of technical parameters, especially the balance of read counts for the allelic pairs. Additional selection criteria were added to the algorithm based on the analysis of approximately 1,000 controlled cross populations. Testing for Mendelian distribution of alleles in these populations facilitated selection of technical parameters discriminating true allelic variants from paralogous sequences. In addition, multiple samples were processed from DNA to allelic calls as technical replicates and scoring consistency was used as the main selection criteria for high quality/low error rate markers. Calling quality was assured by high average read depth per locus (Average across all markers was over 20 reads/locus).

**References**

Altshuler, D., Pollara, V.J., Cowles, C.R., Van Etten, W.J., Baldwin, J., Linton, L., & Lander, E.S. (2000). An SNP map of the human genome generated by reduced representation shotgun sequencing. *Nature,* *407*(6803): 513-516. doi: 10.1038/35035083. PMID: 11029002.

Baird, N.A., Etter, P.D., Atwood, T.S., Currey, M.C., Shiver, A.L., Lewis, Z.A., Selker, E. U., Cresko, W. A. & Johnson,E. A. (2008). Rapid SNP discovery and genetic mapping using sequenced RAD markers. *PLoS ONE*, *3*(10): e3376 https://doi.org/10.1371/journal.pone.0003376

Courtois, B., Audebert, A., Dardou, A., Roques, S., Ghneim-Herrera, T., Droc, G., Frouin, J., Rouan, L., Gozé, E., Kilian, A., Ahmadi, N. & Dingkuhn, M. (2013). Genome-wide association mapping of root traits in a Japonica Rice Panel. *PLoS ONE, 8*(11): e78037. https://doi.org/10.1371/journal.pone.0078037

Cruz, V.M.V., Kilian, A. & Dierig, D.A. (2013). Development of DArT marker platforms and genetic diversity assessment of the U.S. collection of the new oilseed crop *Lesquerella* and related species. *PLoS ONE, 8*(5): e64062. https://doi.org/10.1371/journal.pone.0064062

Elshire, R.J., Glaubitz, J.C., Sun, Q., Poland, J.A., Kawamoto, K., Buckler, E.S. & Mitchell, S. E. (2011). A robust, simple genotyping-by-sequencing (GBS) approach for high diversity species. *PLoS ONE, 6*(5): e19379. https://doi.org/10.1371/journal.pone.0019379

Kilian, A., Wenzl, P., Huttner, E., Carling, J., Xia, L., Blois, H., Caig, V., Heller-Uszynska, K., Jaccoud, D., Hopper, C., Aschenbrenner-Kilian, M., Evers, M., Peng, K., Cayla, C., Hok, P., & Uszynski, G. (2012). Diversity Arrays Technology: A Generic Genome Profiling Technology on Open Platforms. In: Pompanon, F., Bonin, A. (eds) Data Production and Analysis in Population Genomics. Methods in Molecular Biology, vol 888, pp. 67–89. Humana Press, Totowa, NJ. https://doi.org/10.1007/978-1-61779-870-2_5

Raman, H., Raman, R., Kilian, A., Detering, F., Carling, J., Coombes, N., Diffey, S., Kadkol, G., Edwards, D., McCully, M., Ruperao, P., Parkin, I. A., Batley, J., Luckett, D. J., Wratten, N. (2014). Genome-wide delineation of natural variation for pod shatter resistance in *Brassica napus*. *PLoS One, 9*(7):e101673. doi: 10.1371/journal.pone.0101673. PMID: 25006804; PMCID: PMC4090071.

Sansaloni, C., Petroli, C., Jaccoud, D. Carling, J., Detering, F., Grattapaglia, D. & Kilian, A. (2011). Diversity Arrays Technology (DArT) and next-generation sequencing combined: genome-wide, high throughput, highly informative genotyping for molecular breeding of *Eucalyptus*. *BMC Proceedings*, 5(7), P54. https://doi.org/10.1186/1753-6561-5-S7-P54

Sudyka, J., Di Lecce, I., Wojas, L., Rowiński, P. and Szulkin, M. (2022), Nest-boxes alter the reproductive ecology of urban cavity-nesters in a species-dependent way. Journal of Avian Biology, 2022: e03051. https://doi.org/10.1111/jav.03051

**Figure S1 –**
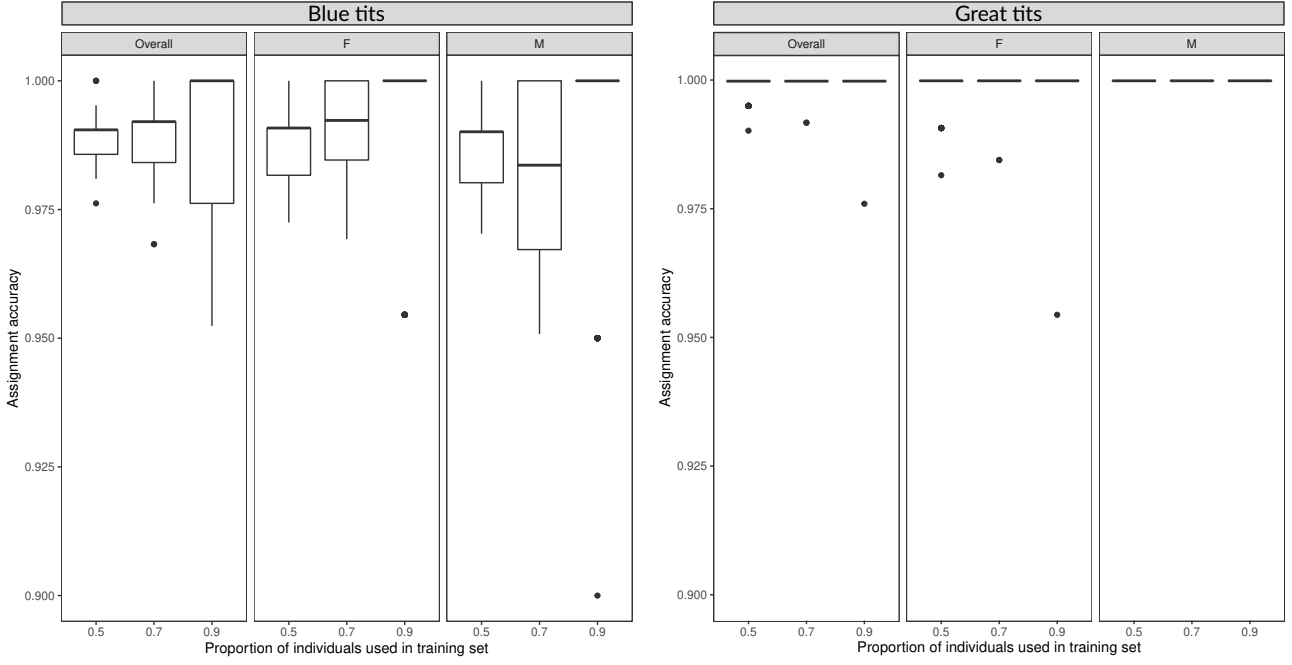
Sex assignment accuracy of Monte-Carlo cross-validation using 50%, 70%, and 90% of individuals from each population (overall: all individuals, F: females, M: males) randomly chosen for the training. Models were run with 100 iterations. Support Vector Machine was used as classification method for the prediction in blue tits and Random forest in great tits. The dataset used to infer sex included all nestlings sampled within Warsaw as part of a larger study on extra-pair paternity (n=3031).

**Table S2 –** Correlation table of spatio-temporal variables for each radius in blue tits and great tits.

|  |  | **Blue tits** | | | | | | **Great tits** | | | | | |
| --- | --- | --- | --- | --- | --- | --- | --- | --- | --- | --- | --- | --- | --- |
|  |  | **96 m** | | **192 m** | | **288 m** | | **72 m** | | **144 m** | | **216 m** | |
|  |  | **r** | **p** | **r** | **p** | **r** | **p** | **r** | **p** | **r** | **p** | **r** | **p** |
| **Nearest neighbour distance vs**  **Neighbour density** | | -0.606 | <0.001 | -0.426 | <0.001 | -0.403 | <0.001 | -0.751 | <0.001 | -0.381 | <0.001 | -0.281 | 0.002 |
| **Nearest neighbour distance vs**  **Synchronous neighbour density** | | -0.534 | <0.001 | -0.466 | <0.001 | -0.431 | <0.001 | -0.465 | <0.001 | -0.252 | 0.006 | -0.183 | 0.050 |
| **Neighbour density vs**  **Synchronous neighbour density** | | 0.701 | <0.001 | 0.702 | <0.001 | 0.670 | <0.001 | 0.673 | <0.001 | 0.681 | <0.001 | 0.731 | <0.001 |

**Table S3 –** Number of broods with number of extra-pair offspring per species and cavity type. Only broods whose parents were caught were included, as the number of extra-pair offspring can be identified comparing relatedness of each nestling to the social father (1 natural cavity and 2 nestboxes were excluded in blue tits and 2 natural cavities and 1 nestbox in great tits).

|  | **Blue tits** | | **Great tits** | |
| --- | --- | --- | --- | --- |
| **Number of extra-pair chick per brood** | **Natural cavity** | **Nestbox** | **Natural cavity** | **Nestbox** |
| 0 | 19 | 20 | 20 | 10 |
| 1 | 6 | 7 | 4 | 5 |
| 2 | 3 | 4 | 3 | 6 |
| 3 | 0 | 3 | 2 | 3 |
| 4 | 1 | 1 | 1 | 1 |
| **Total** | **29** | **35** | **30** | **25** |

**Table S4 –** Spatio-temporal parameters among cavity types when including natural cavities found in the nestbox plot in 2019. In blue tits two cavities and in great tits one cavity outside the core area were removed. Significant differences (P < 0.05) are in bold, trends (P < 0.2) in italics.

|  | **Nearest neighbour distance** | | | |  | **Neighbour density** | | | | **Synchronous neighbour density** | | | |
| --- | --- | --- | --- | --- | --- | --- | --- | --- | --- | --- | --- | --- | --- |
|  | **Wilcoxon rank sum test** | | | |  | **Wilcoxon rank sum test** | | | | **Wilcoxon rank sum test** | | | |
|  | **W** | **P** | **Median in the cavity plot (m)** | **Median in the nestbox plot (m)** | **Radius** | **W** | **P** | **Median number in the cavity plot (range)** | **Median number in the nestbox plot (range)** | **W** | **P** | **Median number in the cavity plot (range)** | **Median number in the nestbox plot (range)** |
| **Blue tits**  **(n = 138)** | 2787 | **0.019** | 58 | 48 | **96 m** | 1414.5 | **<0.001** | 2 (0-7) | 3 (0-6) | 1451.5 | **<0.001** | 1 (0-5) | 2 (0-6) |
|  |  |  |  |  | **192 m** | 1476.5 | **<0.001** | 9 (1-17) | 11 (5-19) | 1640.5 | **0.007** | 4 (0-13) | 6 (0-13) |
|  |  |  |  |  | **288 m** | 1847.5 | *0.076* | 18 (2-31) | 19 (11-29) | 1803.5 | **0.049** | 9 (1-19) | 12 (1-18) |
| **Great tits**  **(n = 126)** | 1499 | 0.41 | 50 | 54 | **72 m** | 1454 | 0.262 | 1 (0-4) | 1 (0-4) | 1435.5 | 0.205 | 0 (0-3) | 1 (0-3) |
|  |  |  |  |  | **144 m** | 1640 | 0.946 | 4 (0-11) | 4.5 (2-8) | 1292.5 | *0.050* | 2 (0-7) | 3 (0-7) |
|  |  |  |  |  | **216 m** | 1826 | 0.353 | 9 (2-21) | 10 (3-15) | 1412.5 | 0.195 | 5 (0-12) | 6 (0-13) |

**Table S5 –** Spatio-temporal parameters for blue and great tits nests among the two study years. Trends (P < 0.2) are in italics.

|  | **Nearest neighbour distance** | | | |  | **Neighbour density** | | | | **Synchronous neighbour density** | | | |
| --- | --- | --- | --- | --- | --- | --- | --- | --- | --- | --- | --- | --- | --- |
|  | **Wilcoxon rank sum test** | | | |  | **Wilcoxon rank sum test** | | | | **Wilcoxon rank sum test** | | | |
|  | **W** | **P** | **Median in 2018 (m)** | **Median in 2019 (m)** | **Radius** | **W** | **P** | **Median number in 2018 (range)** | **Median number in 2019 (range)** | **W** | **P** | **Median number in 2018 (range)** | **Median number in 2019 (range)** |
| **Blue tits (n = 131)** | 2404 | 0.223 | 53 | 50 | **96 m** | 1830.5 | *0.149* | 2 (0-6) | 3 (0-7) | 1990 | 0.254 | 1 (0-6) | 1 (0-5) |
|  |  |  |  |  | **192 m** | 1822.5 | *0.144* | 9 (1-16) | 10 (2-18) | 2005 | 0.536 | 4 (0-13) | 5 (0-13) |
|  |  |  |  |  | **288 m** | 1772.5 | *0.091* | 17 (2-27) | 19 (4-31) | 2057.5 | 0.708 | 9 (1-18) | 10 (1-19) |
| **Great tits (n = 116)** | 1500 | 0.318 | 51 | 57 | **72 m** | 1903 | *0.196* | 1 (0-4) | 1 (0-4) | 1699.5 | 0.916 | 1 (0-3) | 1 (0-3) |
|  |  |  |  |  | **144 m** | 1979.5 | *0.098* | 5 (1-11) | 4 (0-11) | 1971.5 | *0.105* | 3 (0-7) | 2 (0-7) |
|  |  |  |  |  | **216 m** | 2031 | *0.053* | 10 (4-21) | 7 (1-19) | 2019.5 | *0.061* | 6 (0-13) | 5 (0-12) |

**Table S6 -** Generalized linear mixed models for blue tits and generalized linear models for great tits with presence of extra-pair paternity (0/1) in the nest as the dependent variable. Cavity type, laying date, clutch size, neighbour density (model b) and synchronous neighbour density (model c) within 192 and 288 m in blue tits and 144 and 216 m in great tits respectively were included as predictors. Mother identity was introduced as random effect in models for blue tits. Reference levels were: natural cavity (cavity type) and 2018 (year). Significant differences (P < 0.05) are in bold, trends (P < 0.2) in italics.

| **Occurrence of extra-pair paternity** | | | | | |
| --- | --- | --- | --- | --- | --- |
| **Blue tits (n = 52)** | | **192 m** | | **288 m** | |
|  |  | **Estimate (SE)** | **Pr(>χ^2^)** | **Estimate (SE)** | **Pr(>χ^2^)** |
| **Model b** | **Neighbour density** | 0.247 (0.350) | 0.480 | 0.269 (0.328) | 0.413 |
|  | **Cavity type** | 0.374 (0.640) | 0.559 | 0.417 (0.617) | 0.499 |
|  | **Laying date** | 0.061 (0.374) | 0.871 | 0.062 (0.370) | 0.868 |
|  | **Clutch size** | -0.066 (0.200) | 0.742 | -0.052 (0.194) | 0.788 |
| **Model c** | **Synchronous neighbour density** | 0.416 (0.480) | 0.386 | 0.172 (0.507) | 0.735 |
|  | **Cavity type** | 0.085 (0.800) | 0.916 | 0.358 (0.817) | 0.662 |
|  | **Laying date** | 0.266 (0.394) | 0.499 | 0.222 (0.432) | 0.608 |
|  | **Clutch size** | -0.079 (0.206) | 0.699 | -0.036 (0.197) | 0.855 |
| **Great tits (n = 48)** | | **144 m** | | **216 m** | |
|  |  | **Estimate (SE)** | **Pr(>χ^2^)** | **Estimate (SE)** | **Pr(>χ^2^)** |
| **Model b** | **Neighbour density** | 0.209 (0.403) | 0.602 | -0.222 (0.418) | 0.594 |
|  | **Cavity type** | 1.052 (0.761) | *0.158* | 0.727 (0.746) | 0.328 |
|  | **Laying date** | 1.100 (0.469) | **0.008** | 1.038 (0.467) | **0.012** |
|  | **Clutch size** | -0.368 (0.240) | *0.107* | -0.343 (0.233) | *0.126* |
| **Model c** | **Synchronous neighbour density** | 0.278 (0.372) | 0.450 | -0.011 (0.362) | 0.976 |
|  | **Cavity type** | 0.833 (0.694) | 0.228 | 0.896 (0.705) | 0.201 |
|  | **Laying date** | 1.086 (0.466) | **0.008** | 1.082 (0.466) | **0.009** |
|  | **Clutch size** | -0.365 (0.240) | *0.110* | -0.354 (0.234) | *0.115* |

**Table S7** - Generalized linear models for blue tits with presence of extra-pair paternity (0/1) in the nest as the dependent variable. Cavity type, laying date, clutch size, nearest neighbour distance (model a), neighbour density (model b) and synchronous neighbour density (model c) were included as predictors. Reference levels were: natural cavity (cavity type) and 2018 (year). Only fixed effects were included here.

| **Occurrence of extra-pair paternity** | | | | | | | |
| --- | --- | --- | --- | --- | --- | --- | --- |
| **Blue tits (n = 59)** | | | | | | | |
|  | | **Estimate (SE)** | **Pr(>χ^2^)** |  | | | |
| **Model a** | **Nearest neighbour distance** | -0.387 (0.325) | 0.228 |  | | | |
|  | **Cavity type** | -0.073 (0.610) | 0.905 |  |  |  |  |
|  | **Laying date** | -0.065 (0.337) | 0.847 |  | | | |
|  | **Clutch size** | -0.074 (0.184) | 0.687 |  | | | |
|  | | **96 m** | | **192 m** | | **288 m** | |
|  |  | **Estimate (SE)** | **Pr(>χ^2^)** | **Estimate (SE)** | **Pr(>χ^2^)** | **Estimate (SE)** | **Pr(>χ^2^)** |
| **Model b** | **Neighbour density** | 0.210 (0.305) | 0.489 | 0.111 (0.320) | 0.730 | 0.208 (0.300) | 0.483 |
|  | **Cavity type** | 0.094 (0.588) | 0.874 | 0.147 (0.602) | 0.807 | 0.123 (0.574) | 0.830 |
|  | **Laying date** | -0.056 (0.341) | 0.871 | -0.025 (0.340) | 0.943 | -0.042 (0.335) | 0.901 |
|  | **Clutch size** | -0.076 (0.187) | 0.686 | -0.066 (0.193) | 0.733 | -0.072 (0.186) | 0.699 |
| **Model c** | **Synchronous neighbour density** | 0.026 (0.349) | 0.941 | 0.221 (0.430) | 0.607 | -0.062 (0.470 | 0.895 |
|  | **Cavity type** | 0.200 (0.674) | 0.766 | -0.033 (0.754) | 0.966 | 0.302 (0.785) | 0.700 |
|  | **Laying date** | 0.012 (0.333) | 0.972 | 0.075 (0.354) | 0.833 | -0.022 (0.395) | 0.956 |
|  | **Clutch size** | -0.046 (0.186) | 0.806 | -0.078 (0.194) | 0.687 | -0.036 (0.189) | 0.851 |

**Table S8 -** Generalized linear mixed models with proportion of extra-pair offspring per nest as the dependent variable. Cavity type, laying date, clutch size, neighbour density (model b) and synchronous neighbour density (model c) within 192 and 288 m in blue tits and 144 and 216 m in great tits were included as predictors. Mother identity was introduced as random effect. Reference levels were: natural cavity (cavity type) and 2018 (year). Significant differences (P < 0.05) are in bold, trends (P < 0.2) in italics.

| **Proportion of extra-pair offspring per nest** | | | | | |
| --- | --- | --- | --- | --- | --- |
| **Blue tits (n = 50)** | | **192 m** | | **288 m** | |
|  |  | **Estimate (SE)** | **Pr(>χ^2^)** | **Estimate (SE)** | **Pr(>χ^2^)** |
| **Model b** | **Neighbour density** | 0.328 (0.292) | 0.263 | 0.353 (0.272) | *0.194* |
|  | **Cavity type** | 0.230 (0.542) | 0.672 | 0.333 (0.504) | 0.509 |
|  | **Laying date** | -0.027 (0.317) | 0.933 | -0.014 (0.307) | 0.963 |
|  | **Clutch size** | -0.219 (0.165) | *0.184* | -0.203 (0.158) | 0.201 |
| **Model c** | **Synchronous neighbour density** | 0.271 (0.371) | 0.466 | 0.148 (0.423) | 0.726 |
|  | **Cavity type** | 0.160 (0.659) | 0.808 | 0.299 (0.688) | 0.664 |
|  | **Laying date** | 0.151 (0.330) | 0.647 | 0.142 (0.366) | 0.697 |
|  | **Clutch size** | -0.204 (0.170) | 0.231 | -0.178 (0.168) | 0.288 |
| **Great tits (n = 44)** | | **144 m** | | **216 m** | |
|  |  | **Estimate (SE)** | **Pr(>χ^2^)** | **Estimate (SE)** | **Pr(>χ^2^)** |
| **Model b** | **Neighbour density** | -0.050 (0.247) | 0.839 | -0.340 (0.272) | 0.212 |
|  | **Cavity type** | 0.231 (0.509) | 0.650 | 0.049 (0.517) | 0.925 |
|  | **Laying date** | 0.327 (0.240) | *0.173* | 0.269 (0.244) | 0.270 |
|  | **Clutch size** | -0.145 (0.152) | 0.340 | -0.143 (0.155) | 0.358 |
| **Model c** | **Synchronous neighbour density** | 0.044 (0.220) | 0.841 | -0.170 (0.233) | 0.468 |
|  | **Cavity type** | 0.258 (0.485) | 0.595 | 0.354 (0.514) | 0.491 |
|  | **Laying date** | 0.333 (0.243) | *0.170* | 0.303 (0.247) | 0.220 |
|  | **Clutch size** | -0.146 (0.153) | 0.340 | -0.144 (0.157) | 0.358 |

**Table S9** – Generalized linear mixed models for blue tits and generalized linear models for great tits with presence of extra-pair paternity (0/1) in the nest as the dependent variable. Cavity type, year, laying date, clutch size, nearest neighbour distance (model a), neighbour density (model b) and synchronous neighbour density (model c) were included as predictors. Mother identity was introduced as random effect in models for blue tits. Reference levels were: natural cavity (cavity type) and 2018 (year). Significant differences (P < 0.05) are in bold, trends (P < 0.2) in italics.

| **Occurrence of extra-pair paternity** | | | | | | | |
| --- | --- | --- | --- | --- | --- | --- | --- |
| **Blue tits (n = 52)** | | | | | | | |
|  | | **Estimate (SE)** | **Pr(>χ^2^)** |  | | | |
| **Model a** | **Nearest neighbour distance** | -0.014 (0.016) | 0.365 |  |  |  |  |
|  | **Cavity type** | 0.315 (0.669) | 0.637 |  |  |  |  |
|  | **Year** | 0.098 (0.621) | 0.875 |  |  |  |  |
|  | **Laying date** | 0.0239 (0.392) | 0.951 |  |  |  |  |
|  | **Clutch size** | **-**0.055 (0.195) | 0.778 |  |  |  |  |
|  | | **96 m** | | **192 m** | | **288 m** | |
|  |  | **Estimate (SE)** | **Pr(>χ^2^)** | **Estimate (SE)** | **Pr(>χ^2^)** | **Estimate (SE)** | **Pr(>χ^2^)** |
| **Model b** | **Neighbour density** | 0.286 (0.235) | 0.223 | 0.061 (0.087) | 0.482 | 0.0513 (0.062) | 0.410 |
|  | **Cavity type** | 0.310 (0.657) | 0.637 | 0.392 (0.653) | 0.548 | 0.435 (0.629) | 0.489 |
|  | **Year** | 0.187 (0.633) | 0.767 | 0.105 (0.612) | 0.864 | 0.053 (0.623) | 0.932 |
|  | **Laying date** | -0.048 (0.409) | 0.908 | 0.042 (0.392) | 0.914 | 0.037 (0.390) | 0.924 |
|  | **Clutch size** | -0.099 (0.205) | 0.630 | -0.072 (0.202) | 0.721 | -0.059 (0.196) | 0.763 |
| **Model c** | **Synchronous neighbour density** | 0.013 (0.260) | 0.961 | 0.094 (0.125) | 0.452 | 0.025 (0.099) | 0.798 |
|  | **Cavity type** | 0.550 (0.726) | 0.449 | 0.170 (0.805) | 0.833 | 0.423 (0.833) | 0.612 |
|  | **Year** | 0.150 (0.627) | 0.811 | 0.224 (0.630) | 0.722 | 0.193 (0.644) | 0.764 |
|  | **Laying date** | 0.127 (0.378) | 0.737 | 0.227 (0.405) | 0.576 | 0.187 (0.447) | 0.676 |
|  | **Clutch size** | -0.027 (0.199) | 0.892 | -0.081 (0.208) | 0.697 | -0.038 (0.199) | 0.850 |
| **Great tits (n = 48)** | | | | | | | |
|  | | **Estimate (SE)** | **Pr(>χ^2^)** |  | | | |
| **Model a** | **Nearest neighbour distance** | -0.001 (0.016) | 0.937 |  |  |  |  |
|  | **Cavity type** | 0.990 (0.697) | *0.149* |  |  |  |  |
|  | **Year** | 0.580 (0.812) | 0.473 |  |  |  |  |
|  | **Laying date** | 0.895 (0.455) | **0.028** |  |  |  |  |
|  | **Clutch size** | **-**0.330 (0.259) | *0.184* |  |  |  |  |
|  | | **72 m** | | **144 m** | | **216 m** | |
|  |  | **Estimate (SE)** | **Pr(>χ^2^)** | **Estimate (SE)** | **Pr(>χ^2^)** | **Estimate (SE)** | **Pr(>χ^2^)** |
| **Model b** | **Neighbour density** | 0.531 (0.392) | *0.163* | 0.067 (0.148) | 0.651 | -0.051 (0.092) | 0.584 |
|  | **Cavity type** | 1.156 (0.724) | *0.102* | 1.122 (0.758) | *0.129* | 0.789 (0.754) | 0.291 |
|  | **Year** | 0.831 (0.857) | 0.326 | 0.637 (0.825) | 0.437 | 0.414 (0.869) | 0.634 |
|  | **Laying date** | 0.968 (0.475) | **0.022** | 0.892 (0.456) | **0.030** | 0.883 (0.451) | **0.031** |
|  | **Clutch size** | -0.312 (0.259) | 0.207 | -0.336 (0.261) | *0.175* | *-*0.329 (0.259) | *0.186* |
| **Model c** | **Synchronous neighbour density** | 0.845 (0.500) | *0.083* | 0.153 (0.187) | 0.407 | -0.003 (0.110) | 0.978 |
|  | **Cavity type** | 0.790 (0.726) | 0.277 | 0.945 (0.688) | *0.166* | 0.981 (0.690) | *0.151* |
|  | **Year** | 0.884 (0.859) | 0.296 | 0.637 (0.817) | 0.432 | 0.571 (0.858) | 0.505 |
|  | **Laying date** | 1.075 (0.510) | **0.015** | 0.862 (0.455) | **0.036** | 0.898 (0.453) | **0.027** |
|  | **Clutch size** | -0.271 (0.249) | 0.258 | -0.343 (0.261) | *0.167* | *-*0.331 (0.259) | *0.181* |

**Table S10** – Generalized linear mixed models with proportion of extra-pair offspring per nest as the dependent variable. Cavity type, year, laying date, clutch size, nearest neighbour distance (model a), neighbour density (model b) and synchronous neighbour density (model c) were included as predictors. Mother identity was introduced as random effect. Reference levels were: natural cavity (cavity type) and 2018 (year). Significant differences (P < 0.05) are in bold, trends (P < 0.2) in italics.

| **Proportion of extra-pair offspring per nest** | | | | | | | |
| --- | --- | --- | --- | --- | --- | --- | --- |
| **Blue tits (n = 50)** | | | | | | | |
|  | | **Estimate (SE)** | **Pr(>χ^2^)** |  | | | |
| **Model a** | **Nearest neighbour distance** | -0.021 (0.014) | *0.128* |  |  |  |  |
|  | **Cavity type** | 0.065 (0.552) | 0.906 |  |  |  |  |
|  | **Year** | -0.226 (0.506) | 0.655 |  |  |  |  |
|  | **Laying date** | -0.106 (0.345) | 0.758 |  |  |  |  |
|  | **Clutch size** | **-**0.190 (0.154) | 0.218 |  |  |  |  |
|  | | **96 m** | | **192 m** | | **288 m** | |
|  |  | **Estimate (SE)** | **Pr(>χ^2^)** | **Estimate (SE)** | **Pr(>χ^2^)** | **Estimate (SE)** | **Pr(>χ^2^)** |
| **Model b** | **Neighbour density** | 0.362 (0.170) | **0.033** | 0.081 (0.072) | 0.259 | 0.072 (0.051) | *0.157* |
|  | **Cavity type** | 0.038 (0.534) | 0.944 | 0.193 (0.554) | 0.727 | 0.294 (0.509) | 0.564 |
|  | **Year** | -0.060 (0.506) | 0.906 | -0.194 (0.518) | 0.709 | -0.250 (0.514) | 0.627 |
|  | **Laying date** | -0.126 (0.333) | 0.706 | -0.080 (0.346) | 0.818 | -0.079 (0.335) | 0.815 |
|  | **Clutch size** | -0.226 (0.152) | *0.136* | -0.225 (0.163) | *0.168* | -0.213 (0.156) | *0.173* |
| **Model c** | **Synchronous neighbour density** | 0.033 (0.217) | 0.879 | 0.056 (0.097) | 0.564 | 0.021 (0.082) | 0.799 |
|  | **Cavity type** | 0.393 (0.630) | 0.533 | 0.208 (0.661) | 0.753 | 0.328 (0.696) | 0.638 |
|  | **Year** | -0.080 (0.523) | 0.878 | -0.053 (0.523) | 0.919 | -0.049 (0.543) | 0.928 |
|  | **Laying date** | 0.034 (0.337) | 0.920 | 0.089 (0.352) | 0.801 | 0.082 (0.389) | 0.833 |
|  | **Clutch size** | -0.174 (0.164) | 0.290 | -0.207 (0.171) | 0.228 | -0.182 (0.168) | 0.278 |
| **Great tits (n = 44)** | | | | | | | |
|  | | **Estimate (SE)** | **Pr(>χ^2^)** |  | | | |
| **Model a** | **Nearest neighbour distance** | 0.011 (0.012) | 0.364 |  |  |  |  |
|  | **Cavity type** | 0.447 (0.549) | 0.415 |  |  |  |  |
|  | **Year** | 0.714 (0.530) | *0.178* |  |  |  |  |
|  | **Laying date** | 0.209 (0.263) | 0.427 |  |  |  |  |
|  | **Clutch size** | **-**0.074 (0.172) | 0.666 |  |  |  |  |
|  | | **72 m** | | **144 m** | | **216 m** | |
|  |  | **Estimate (SE)** | **Pr(>χ^2^)** | **Estimate (SE)** | **Pr(>χ^2^)** | **Estimate (SE)** | **Pr(>χ^2^)** |
| **Model b** | **Neighbour density** | -0.088 (0.283) | 0.756 | -0.024 (0.102) | 0.811 | -0.086 (0.067) | 0.200 |
|  | **Cavity type** | 0.551 (0.548) | 0.315 | 0.498 (0.571) | 0.383 | 0.232 (0.588) | 0.692 |
|  | **Year** | 0.7462 (0.536) | *0.164* | 0.722 (0.521) | *0.166* | 0.528 (0.543) | 0.331 |
|  | **Laying date** | 0.188 (0.264) | 0.477 | 0.193 (0.259) | 0.456 | 0.173 (0.264) | 0.513 |
|  | **Clutch size** | -0.072 (0.174) | 0.681 | -0.077 (0.169) | 0.647 | *-*0.059 (0.175) | 0.734 |
| **Model c** | **Synchronous neighbour density** | 0.127 (0.300) | 0.671 | 0.038 (0.123) | 0.759 | -0.062 (0.078) | 0.427 |
|  | **Cavity type** | 0.475 (0.545) | 0.383 | 0.541 (0.539) | 0.316 | 0.618 (0.565) | 0.274 |
|  | **Year** | 0.672 (0.516) | *0.193* | 0.709 (0.520) | *0.172* | 0.616 (0.543) | 0.257 |
|  | **Laying date** | 0.194 (0.252) | 0.442 | 0.180 (0.261) | 0.491 | 0.181 (0.265) | 0.494 |
|  | **Clutch size** | -0.088 (0.163) | 0.588 | -0.086 (0.169) | 0.610 | *-*0.062 (0.176) | 0.724 |
